# Factors influencing readiness for advance care planning in dementia: a qualitative interview study

**DOI:** 10.1186/s12904-026-02012-4

**Published:** 2026-02-09

**Authors:** Vera van der Nulft, Arianne Stoppelenburg, Liselotte A. I. Mahieu, Hinke E. Hoffstädt, Jenny T. van der Steen, Liesbeth M. van Vliet, Yvette M. van der Linden

**Affiliations:** 1 Center of Expertise in Palliative Care, Leiden University Medical Center, Albinusdreef 2, Leiden, 2333 The Netherlands; 2 Department of Public Health and Primary Care, Leiden University Medical Center, Leiden, The Netherlands; 3 Radboudumc Alzheimer Center and Department of Primary and Community Care, Radboud university medical center, Nijmegen, the Netherlands; 4 Cicely Saunders Institute, King’s College London, London, United Kingdom; 5 Health, Medical and Neuropsychology Unit, Leiden University, Leiden, The Netherlands

**Keywords:** Alzheimer’s disease, Advance care planning, Communication, Dementia, End of life care, Palliative care, Patient-centered care, Shared decision making, Qualitative research

## Abstract

**Background:**

Despite its recognized importance in dementia care, advance care planning is frequently postponed. Understanding factors that support and hinder readiness (i.e., an individual’s willingness and ability to engage in advance care planning discussions) is essential, to ensure that people with dementia and their family caregivers can participate effectively in planning for future care. This study aimed to identify factors influencing readiness from the perspectives of people with dementia, family caregivers, and healthcare professionals.

**Methods:**

Semi-structured qualitative interviews were conducted with people with dementia, family caregivers, and healthcare professionals across multiple care settings in the Netherlands. Inductive qualitative content analysis was used to identify key factors influencing readiness.

**Results:**

For people with dementia (*n* = 6), readiness was facilitated by a desire for autonomy. Readiness was hindered by focusing on the present, surrendering to a perceived lack of control over the future, avoiding sensitive topics, and relying on healthcare professionals to initiate discussions. For both people with dementia and family caregivers (*n* = 5 family caregivers), readiness was facilitated by a desire to prepare for the future, and hindered by the complexity of advance care planning. (Lack of) collaboration and social support, previous personal experiences, and the relationship with the healthcare professional either facilitated or hindered readiness. No distinct factors were identified solely for family caregivers. Healthcare professionals (*n* = 13) reported factors related to individual healthcare professionals: difficulty addressing sensitive topics as hindering readiness for advance care planning, while (lack of) personal priority, (limited) education and training, and (lack of) experience either facilitated or hindered it. They also reported factors related to healthcare organizations: interprofessional cooperation and communication facilitated readiness, while (lack of) implementation leadership, (limited) time to implement advance care planning, and (limited) procedural support either facilitated or hindered readiness.

**Conclusions:**

Readiness for advance care planning in dementia is dynamic and shaped by personal, relational, and systemic factors. Addressing these factors may facilitate timely and meaningful implementation of advance care planning, ensuring future care aligns with the values and preferences of people with dementia and their families.

**Supplementary Information:**

The online version contains supplementary material available at 10.1186/s12904-026-02012-4.

## Background

People with dementia can experience significant challenges in decision making and communication, making it difficult to elicit wishes and needs for future care and treatment, especially in a later stage of the disease [1, 2]. Given the symptoms and the progressive nature of dementia, initiating advance care planning (ACP) at an early stage of the disease is essential to facilitate informed shared decision making regarding the future [3]. ACP involves the process of identifying values, goals and preferences for future care and treatment between the person with dementia, their family caregivers and healthcare professionals [4]. This may help people with dementia in retaining autonomy and control over their lives as their cognitive capacity declines over time [5–7]. Timely engagement in ACP also provides guidance and support for family caregivers, who gradually assume a more central role in decision making as the cognitive capacity of the person with dementia declines. Despite its recognized importance within the context of dementia, research indicates that ACP is frequently postponed or, in some cases, not undertaken at all [8–11].

Reasons for limited participation in ACP among people with dementia and family caregivers are multifaceted. ACP topics can be emotionally distressing, as some individuals experience strong emotional responses when confronted with end-of-life issues, leading to reluctance to think about or discuss the prognosis or future care [12, 13]. ACP is often considered non-urgent by both people with dementia and family caregivers when the current condition still feels manageable, which contributes to postponing such discussions at present [12, 14, 15]. This attitude can be further compounded by denial, as people with dementia may struggle to acknowledge their diagnosis and the implications of its progressive nature [1, 14, 16–19]. Some people with dementia may also view ACP as unnecessary or irrelevant, adopting a day-to-day attitude that deprioritizes long-term considerations [13, 15, 16, 20].

The limited implementation of ACP is also attributed to challenges experienced by healthcare professionals. The most prominent challenges are insufficient education and training in ACP and time constraints, which leaves many healthcare professionals feeling unprepared to facilitate ACP [1, 21–26]. Experiencing discomfort discussing end of life themselves or concern about causing emotional distress in patients and family caregivers, also prevents healthcare professionals from initiating ACP [1, 20–22, 27, 28]. To enable healthcare professionals to start ACP discussions, they need adequate communication skills and experience to address sensitive or difficult topics related to ACP, such as the end of life [12].

For people with dementia and their family caregivers to engage meaningfully in ACP, it is crucial that they experience a supportive relationship with healthcare professionals. People with dementia and family caregivers express a need to trust their healthcare professionals to communicate openly during ACP discussions [29]. A supportive relationship with healthcare professionals and experiencing trust helps people with dementia and family caregivers to feel prepared and willing to participate in ACP discussions. This closely relates to ‘readiness’, an important concept in the context of ACP, encompassing the willingness and ability to engage in ACP conversations [30, 31]. It has been defined as a prerequisite for ACP to initiate the process of ACP, and also as an outcome of participating in ACP itself [32]. This dual aspect of readiness highlights the complex, iterative nature of ACP, and underscores the importance of fostering readiness for both initiation and maintenance of ACP discussions.

Despite growing interest in ACP for people with dementia, readiness for ACP has received limited attention. The aim of the current study is to identify factors that influence readiness for ACP in dementia, from the perspectives of people with dementia, their family caregivers, and healthcare professionals who provide care and support for people with dementia.

## Methods

### Study design

The current study is part of a larger study aimed at improving readiness for ACP of people with dementia, family caregivers, and healthcare professionals, and the use of conversation aids (the READY project) [33]. A qualitative interview design was adopted to identify factors influencing readiness for ACP among people with dementia, their family caregivers, and healthcare professionals providing care for people with dementia. The study design was guided by a constructionist perspective, focusing on understanding participants’ subjective experiences [34, 35]. Consolidated criteria for reporting qualitative research (COREQ) were used as a reporting guideline [36].

### Participant recruitment

The inclusion and exclusion criteria for each participant group are presented in Table 1. Participants were recruited across different care settings in the Netherlands through multiple methods. Recruitment primarily occurred through newsletters distributed within patient and healthcare professional networks. Those interested in participating after receiving the newsletter contacted the interviewer, who followed up via mail or telephone to schedule an appointment. Some participants were recruited via network-based recruitment, where acquaintances or colleagues of the research team were asked to either participate themselves or help identify potential participants. If participants agreed, the interviewer then reached out via mail or telephone to arrange the interview. The interviewer (VN), though part of the research team, had no prior relationship with any of the participants. We used purposive sampling to recruit people with dementia and their family caregivers, aiming for variation in dementia type and stage, and healthcare professionals from diverse disciplines and care settings. As recruitment progressed, we purposefully sought additional participants with underrepresented characteristics to further enhance maximum variation. All participants were informed about the aim of the study and provided informed consent prior to the interview.

**Table 1:** Inclusion and exclusion criteria for each group

**INCLUSION CRITERIA**
**People with dementia:**
- Have received a dementia diagnosis by a general practitioner or medical specialist;
- Receive or have received care from a general practice, home care organization, hospital, or nursing home;
- Are sufficiently proficient in the Dutch language;
- Must have a representative who will provide informed consent on their behalf and accompany them to the interview, in case of limited decision making capacity.
**Family caregivers:**
- Are family members, friends, or neighbors of a person with dementia;
- Care for or support the person with dementia (i.e., provides practical and/or emotional support at least once a week; e.g., assisting with daily activities and medical appointments);
- Are sufficiently proficient in the Dutch language;
- Are 18 years or older.
**Healthcare professionals:**
- Have at least one year of experience as a healthcare or social care professional;
- Work at a general practice, home care organization, hospital, or nursing home in the Netherlands;
- Have regular contact with patients with dementia, with a minimum of at least once a month;
- Are sufficiently proficient in the Dutch language;
- Are 18 years or older.
**EXCLUSION CRITERIA**
Potential participants were excluded if they had severe physical or mental health conditions that would significantly interfere with participation in the study.

### Data collection

Interviews were conducted from January to April 2024 at locations that the participants considered to be most convenient for them. No repeat interviews were conducted. An interview guide was iteratively developed with the study team (see Appendix A). Consistent with a constructionist stance, the interview guide was designed to allow flexibility during the interviews, allowing to explore participants’ specific prior experiences as they arise in the conversation. The interviews consisted of the following topics: (1) Experiences with or expectations of ACP, (2) factors that could improve readiness for ACP, with participants from all groups reflecting on factors related to both healthcare professionals and to people with dementia and family caregivers, and (3) perspectives on using conversation aids. The analyses focused on experiences or expectations of ACP and factors that could improve readiness. The section of the interviews that referred to perspectives on conversation aids was not used in the current analysis, as this was not relevant to the research question for this article. Semi-structured interviews were conducted and audio recorded by a PhD student (VN) with a background in psychology. She received formal training in qualitative interviewing and analyses, which shaped her approach to data collection and encouraged a reflective, open stance in interviewing. Participants were informed of the researcher’s background and goals. Prior to each interview, participants completed a brief questionnaire on sociodemographic characteristics and their experience with dementia and ACP. ACP was explained to each participant as: “having discussed goals, preferences, and choices for future care and support”. Field notes were taken during and after each interview to facilitate monitoring of data saturation and to offer contextual clarification in case transcripts required additional interpretation. Data collection continued until saturation was reached, defined as the point at which no new topics emerged [37], as assessed through ongoing team discussions post-interview summaries. Interviews were transcribed verbatim. Transcripts were not returned to participants for comment or correction.

To enhance the quality of the findings, we applied the four key criteria of trustworthiness in qualitative data: credibility, dependability, confirmability, and transferability [38]. Credibility was ensured through exploration and integration of multiple perspectives across the three groups of participants, to enhance the depth of the findings. Dependability and confirmability were addressed through reflexive journaling and ongoing discussions within the research team, ensuring that the findings were rooted in the data and not influenced by personal biases. Transferability was supported by providing rich contextual details to allow for comparisons in similar settings or participant groups.

### Data analysis

An inductive qualitative content analysis approach was used to develop overarching categories that conceptually represented the data [39, 40]. Content analysis was chosen because it allows for categorization while accommodating interpretive abstraction, making it well-suited for capturing both explicit statements and underlying meanings across the diverse participant perspectives. This approach is useful in exploring the topic of readiness for ACP, where participants may express both clear intentions and deeper, more implicit factors that influence their attitudes and behaviors.

Both manifest and latent coding were applied across all interviews. The transcripts were coded inductively by two researchers (VN & LM) using Atlas.ti (Version 9.0.24). To support consistency of coding and enhance the depth of analysis, two interviews were independently coded by both researchers. The codes were discussed to reach consensus and to collaboratively interpret the data. The remaining interviews were coded by one researcher (VN), with regular consultation with the research team to reflect on findings and to create overarching categories. Codes were synthesized into subcategories and main categories through team discussions, with the final categories being collaboratively drafted by the whole research team. Participants were not asked to provide feedback on the study findings.

## Results

### Participant characteristics

Twenty-four semi-structured interviews were conducted. Six people with dementia, five family caregivers (without the person with dementia present), and thirteen healthcare professionals participated. Two participating people with dementia were accompanied by their spouse during their interview. Although the spouses sometimes expressed their own views on ACP and supported the person with dementia by clarifying statements, only the responses of the people with dementia were analyzed. No participants withdrew from the study.

Interview duration ranged between 21 and 99 min, with an average duration of 59 min. The interviews were held at participants’ homes (*n* = 10), at the workplaces of healthcare professionals (*n* = 9), at a library (*n* = 1), at a communal meeting place for people with memory problems (*n* = 2), and at the research team’s workplace (*n* = 2). The mean ages of participants were 72 years for people with dementia (range 62–79), 68 years for family caregivers (ranging from 54 to 80), and 46 years for healthcare professionals (ranging from 32 to 56). Table 2 outlines sociodemographic characteristics of people with dementia and family caregivers, and Table 3 outlines those of healthcare professionals.

**Table 2:** Characteristics of people with dementia and family caregivers (*N* = 11)

Characteristics	People with dementia (*n* = 6)	Family caregivers (*n* = 5)
	*n*	
Sex		
Female	4	2
Male	2	3
Age		
50–59 years	0	2
60–69 years	1	0
70–79 years	5	2
80–89 years	0	1
Education level		
Primary education	1	0
High school education	2	2
Higher education	3	3
Ethnicity		
Dutch	6	5
Western migration background	0	0
Non-western migration background	0	0
Paid employment		
Yes	0	4
No	6	1
Living situation of person with dementia		
Home, with partner	4	2
Home, alone	1	1
Care institution	1	2
Dementia diagnosis		
Alzheimer	3	1
Frontotemporal	0	1
Lewy body	1	0
Vascular	0	0
Mixed	2	1
Unknown	0	2
Years since diagnosis		
< 1 year	3	0
1–2 years	1	2
> 3 years	1	1
Unknown	1	2
Care setting where receiving care		
Primary care	0	0
Home care organization	3	2
Hospital	1	0
Nursing home	1	2
Day care center	0	1
None	1	0
Family caregiver relationship^a^		
Spouse	3	2
Child	1	2
Friend	1	0
Other^b^	1	1

^a^For participants with dementia, information was collected regarding who their primary family caregiver most involved in their care was. Family caregivers were asked to indicate their relationship to their loved one with dementia. People with dementia and family caregivers participated independently (i.e., no patient-caregiver dyads were included)

^b^One family caregiver was a sister in law, one person with dementia listed their case manager as their primary family caregiver

**Table 3:** Characteristics of healthcare professionals (*N* = 13)

Characteristics	*n*
Sex	
Female	11
Male	2
Age	
30–39 years	4
40–49 years	4
50–59 years	5
Ethnicity	
Dutch	12
Western migration background	1
Non-western migration background	0
Care setting	
Primary care	1
Home care organization	4
Hospital	4
Nursing home	4
Profession	
Case manager or community nurse	3
Nursing staff	2
Nurse practitioner	2
Internal medicine physician	1
Elderly care physician	1
Psychogeriatric care physician^a^	1
Resident	1
Psychiatrist	1
Neurologist	1
Time in current professional position	
< 5 years	2
6–10 years	4
11–19 years	4
> 20 years	3
Dementia case load per month	
< 5 patients	3
6–10 patients	2
11–15 patients	2
16–19 patients	0
> 20 patients	6

^a^In the Netherlands, a psychogeriatric care physician is a general practitioner who has completed a two-year postgraduate training in psychogeriatrics, focusing on the care of older adults with cognitive and psychiatric conditions

Among people with dementia, five had previous work experience in healthcare and five had previous personal exposure to dementia outside of their current situation. Four had previously discussed ACP with a healthcare professional since their diagnosis. Among family caregivers, two reported work experience in healthcare, three had prior personal exposure to dementia, and four had prior ACP discussions. Among healthcare professionals, 10 reported prior professional experience conducting ACP discussions with people with dementia and their family caregivers, and 10 had personal experience with a close contact who had advanced dementia and had passed away.

### Factors influencing readiness for ACP in dementia

**Fig. 1:**
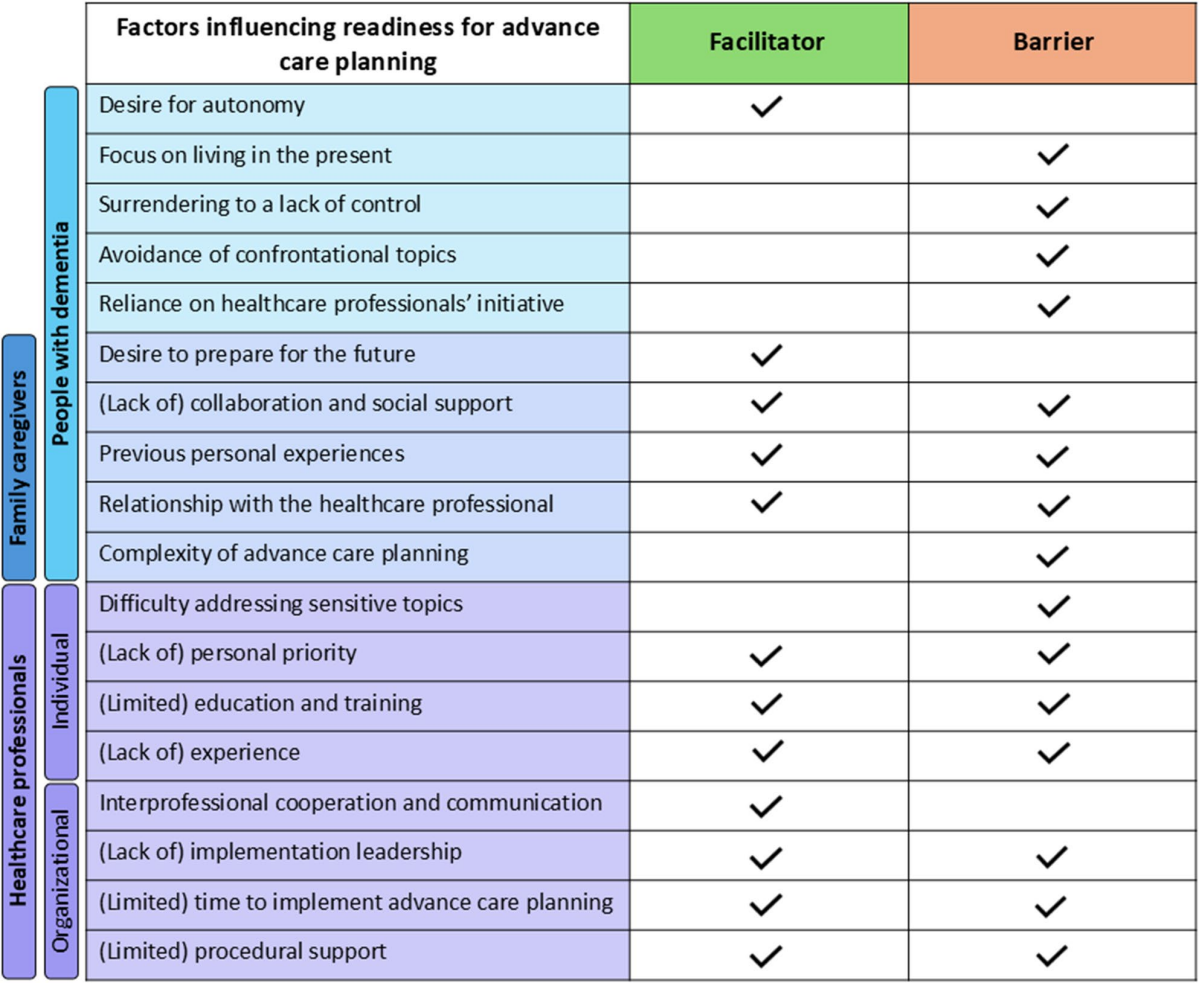
Overview of factors influencing readiness for advance care planning among people with dementia, family caregivers, and healthcare professionals

The analysis identified 18 factors influencing readiness for ACP across the three groups of participants, as shown in Fig. 1. The coding tree with the categories and codes is presented in Appendix B.

### Factors influencing readiness relating only to people with dementia

Analysis yielded five factors influencing readiness for ACP uniquely reported by people with dementia. A *desire for autonomy* facilitated readiness, while *a focus on living in the present*, *surrendering to a lack of control*, *avoidance of confrontational topics*, and *reliance on healthcare professionals’ initiative* hindered readiness.

#### Desire for autonomy

People with dementia often expressed a desire to retain control over important decisions while still capable, enhancing their readiness and motivating early engagement in ACP: *I want to retain some control*,* and I am willing to fight for it.”* (P4, person with Alzheimer’s Disease). ACP was seen as a tool to express wishes and boundaries, to provide guidance to family caregivers and healthcare professionals in the future and ensuring that care decisions remain aligned with the values and preferences of the person with dementia.

#### Focus on living in the present

Some people with dementia preferred to live in the present rather than thinking about the future, which lowered their readiness to engage in ACP: “*There’s no point in thinking about it or worrying about it.*” (P2, person with Alzheimer’s Disease). This perspective affected readiness for ACP, though some present-focused participants still discussed future care with family or healthcare professionals or completed advance directives. Others with this perspective saw ACP as unhelpful, found thinking about the future too abstract or emotionally burdensome, or preferred to leave decisions to family. Some participants explicitly explained their decision to avoid ACP as a way of staying positive: “*You shouldn’t die before you’re in your coffin.*” (P5, person with mixed dementia).

#### Surrendering to a lack of control

Surrendering to a perceived lack of control hindered readiness for ACP among people with dementia. Some viewed the future as unpredictable and beyond their control, actively choosing not to engage in ACP and often viewing it as futile or irrelevant: “*On the other hand*,* I understand that I have a problem that is probably going to get worse over time*,* but I don’t know how to assess it or what exactly that will entail. So I’m not going to think about that*.” (P6, person with Alzheimer’s Disease). Others with this view were hesitant to engage because they were aware that their wishes and preferences would potentially change over time, making current decisions feel uncertain or premature. Participants described this perspective as a reflection of their personality, noting that planning ahead did not fit with the way they generally live their lives.

#### Avoidance of confrontational topics

A key factor hindering readiness for ACP was avoiding emotionally distressing topics related to dementia progression and end of life care. Some participants expressed a clear awareness of this emotional burden, acknowledging that they were not ready to be confronted with specific topics at that time, but expected to talk about those when they would feel emotionally ready for that: “*One moment you’re healthy*,* and the next you have a serious illness. Suddenly we have to talk about death as well. Well*,* that may be*,* but not everything at once*,* and at my own reasonable pace.*” (P2, person with Alzheimer’s Disease).

#### Reliance on healthcare professionals’ initiative

Some people with dementia reported that their readiness to engage in ACP was limited by their reliance on healthcare professionals to initiate ACP discussions, taking a passive role and expecting professionals to lead: “*I actually thought that [my GP] would have called me by now to ask ‘how are you’ or ‘how are you coping’*,* but that hasn’t happened.*” (P2, person with Alzheimer’s Disease). This reliance was also linked to awareness of healthcare professionals’ time constraints, with participants expressing reluctance to burden healthcare professionals while the person was still able to take care of themselves: “*I get the impression that they are always very busy*,* the nursing staff. And besides*,* I can still manage on my own*.” (P1, person with Lewy Body Disease).Most people with dementia included in this study had prior professional experience in healthcare, which contributed to their awareness of potential time constraints.

### Factors influencing readiness relating to people with dementia and family caregivers

Five factors influencing readiness for advance care planning were identified both in interviews of people with dementia and interviews with family caregivers. No distinct factors were identified solely for family caregivers. A *desire to prepare* for the future facilitated readiness. *(A lack of) collaboration and social support*,* previous personal experiences*, and *the relationship with the healthcare professional* either facilitated or hindered readiness, and the *complexity of ACP* hindered readiness.

#### Desire to prepare for the future

A key factor facilitating readiness for ACP among people with dementia and family caregivers was a strong desire to prepare for the future due to the progressive decline of dementia. Both groups often indicated that this stemmed from wanting to minimize future uncertainty and to gain “*some peace of mind*” (P3, person with mixed dementia) by discussing and documenting decisions. Awareness of dementia progression and loss of decision-making capacity in later stages created urgency to plan for the future: “*Fact is: you may be forgetful*,* but you are still competent. And I think there is still time to record it now. At some point*,* and [when] things get worse and people decide you are no longer competent*,* you will be too late to document what you want when things aren’t going well*.” (P6, person with Alzheimer’s Disease). Additionally, readiness was increased for people with dementia who wished to ease family caregivers’ burdens by clarifying preferences in advance. Family caregivers also reported increased readiness for timely documentation of future care preferences and appointment of proxies to ensure proper representation in the future.

#### Collaboration and social support

Collaboration between people with dementia and family caregivers and social support enhanced readiness for ACP, whereas limited collaboration or social support hindered it. For example, both people with dementia and family caregivers valued discussing ACP topics with each other in an informal setting (e.g., at home). They saw ACP as a shared process requiring mutual support. Consequently, some family caregivers faced challenges when the person with dementia was unable to actively participate in ACP, leading to emotional and ethical difficulties when making choices: “*But for me that was the difficult thing. You have to make arrangements for someone else who is not able to oversee the situation at that moment*.” (F5, family caregiver of person with mixed dementia). In hindsight, many of these family caregivers regretted not having started ACP earlier. Additionally, people with dementia and family caregivers valued a broader supportive social network, noting that discussing experiences of dementia and making future care decisions with individuals beyond the immediate family was helpful for understanding and engaging in ACP. For family caregivers, talking about dementia or ACP with friends, other relatives, or support groups made them feel supported. However, some people with dementia reported a lack of support, leading to feelings of isolation.

#### Previous personal experiences

Previous personal and professional experiences with dementia or end of life shaped participants’ readiness either positively or negatively. Many drew on their own or other people’s past experiences to guide their own ACP process. For both people with dementia and family caregivers, stories of negative experiences often acted as motivators to engage in ACP, highlighting undesirable outcomes and thereby prompting earlier action: “*Then I heard things like*,* ‘Yes*,* before you know it*,* you’re in a situation … there is nothing you can do.’ So I thought*,* ‘Oh*,* I’d better make arrangements quickly*.” (P1, person with Lewy Body Disease). Conversely, some people with dementia found that previous negative experiences made them more hesitant to engage in ACP. This included professional experiences that shaped pessimistic expectations about dementia care or concerns about burdening healthcare professionals, as well as distressing encounters with end of life or poor-quality nursing home care.

#### Relationship with the healthcare professional

Trust and the quality of the relationship and interactions with healthcare professionals, when experienced as positive, enhanced readiness. Conversely, low trust, poor relationship quality, and negative interactions with healthcare professionals reduced readiness to engage in ACP: “*I don’t have a very good relationship with the [GP]*,* not at all. And then I think: ‘It would be very unpleasant to have to discuss these things with them’*.” (P1, person with Lewy Body Disease). A trusting or more long-lasting relationship with their main healthcare professional helped people with dementia and family caregivers feel more open to discussing ACP topics. Family caregivers also emphasized the importance of shared decision making, especially in later stages of dementia.

#### Complexity of advance care planning

ACP was viewed as a complex process by people with dementia and family caregivers, covering multiple topics and a range of future care choices, which often hindered readiness. This complexity often led to a limited or incorrect understanding of the purpose of ACP, creating reluctance to seek information or initiate planning, particularly when ACP was viewed as a one-time and irreversible decision. Those who had engaged in ACP mostly credited previous experiences, information provided by healthcare professionals or peers, and educational resources from support networks for improving their readiness. However, many struggled to find clear, consistent information due to fragmented sources: “*Even if I wanted to*,* I don’t know who to turn to to get [information on ACP]. I have no idea*.” (P3, person with mixed dementia).

### Factors influencing readiness relating to individual healthcare professionals

Healthcare professionals identified four factors relating to readiness for ACP at the individual healthcare professional level. *Difficulty addressing sensitive topics* hindered readiness for ACP. *Personal priority*, *education and training*, and *experience* either hindered or facilitated readiness.

#### Difficulty addressing sensitive topics

Healthcare professionals’ readiness for ACP was hindered by difficulties in addressing sensitive topics. Healthcare professionals often found addressing sensitive ACP topics (e.g., end-of-life issues or prognosis) challenging, as these discussions can cause discomfort for everyone involved. Such conversations would demand high sensitivity, empathy, and advanced communication skills, which would require specific training or experience. Healthcare professionals struggled to judge when to raise these topics without causing distress: *“Well*,* sometimes it is difficult*,* where you think: ‘Should I bring this up now? Or will that stir up too much?’.”* (H8, nurse in nursing home). Managing distress while maintaining support was seen as difficult, and many healthcare professionals wished for clearer guidance on how to approach this. Emotionally charged discussions were also reported to sometimes affect healthcare professionals personally, although this did not make them less inclined to deliver ACP: “*If someone tells me difficult things and I shed a tear*,* then so be it.”(H9*,* case manager in home care)*.

#### Personal priority

Healthcare professionals’ readiness for ACP was enhanced when they personally prioritized it, but was reduced when ACP was not seen as a personal priority. Those who perceived ACP as important reported to invest more effort and time into addressing it. Without formal leadership, ACP was reported to depend mostly on individual initiative, with motivated healthcare professionals proactively driving conversations despite competing demands. However, some felt isolated as the sole advocates of ACP within their teams. Those who saw ACP as less urgent than other care tasks argued that engaging in ACP would be on top of other tasks that need to be done regardless: “*These are things that take extra time and effort on top of the usual tasks. Yes*,* I think there’s some laziness too. We’re happy when we get our regular work done. Then [ACP] is another thing on top of that.”* (H11, nursing aide in home care).

#### Education and training

Healthcare professionals consistently emphasized that adequate knowledge and structured training supported readiness to engage in ACP, whereas insufficient education and training limited it. Education and communication skills training were described as increasing confidence and deepening understanding of the goals and processes of ACP. Sufficient knowledge of dementia, disease progression and care options across the disease trajectory was also seen as essential for engaging in ACP and facilitating the process. While practical and medical aspects of ACP were often well-covered, quality of life topics, including existential issues and spiritual wellbeing, were frequently overlooked in formal education: *“Resuscitation is often clear*,* right? But the conversation about quality of life*,* you have to… […] Medicine in particular is still very hospital oriented… [That] is not a standard part of the educational program.”* (H9, psychogeriatric care physician in nursing home).

#### Experience

Besides education and training, healthcare professionals indicated that practical experience with ACP enhanced their readiness, while limited experience reduced it. Greater experience enhances familiarity, communication skills, and confidence, enabling better adaptation of discussions to individual needs: *“And once you’ve done it a few times*,* you realize it’s not that scary at all. So then it becomes much easier and more natural.”* (H5, nurse practitioner in hospital). Repeated practice is also crucial for understanding what ACP discussions should entail: *“And also just knowing: what things are really important to discuss? So that you go through the process from A to Z. And then it’s mainly experience. And you need to practice that a few times*,* I think.”* (H13, resident in general practice). Limited experience was viewed to hinder ACP readiness, with less exposed healthcare professionals expressing uncertainty and a need for more practice opportunities.

### Factors influencing readiness relating to healthcare organizations

Analysis of healthcare professionals’ interviews yielded four factors influencing readiness for ACP relating to the healthcare organization level. *Interprofessional cooperation and communication* facilitated readiness. (A *lack of) implementation leadership*, *(limited) time to implement ACP*, and *(limited) procedural support* either facilitated or hindered readiness for ACP.

#### Interprofessional cooperation and communication

Healthcare professionals highlighted that both transmural and intramural cooperation and communication are crucial for improving ACP readiness amongst healthcare professionals. Collaboration and systematic electronic documentation of ACP conversations were reported to support this process. Interprofessional communication can help to address difficult ethical or emotional dilemmas that may emerge during ACP, and can improve the ACP process when transfer to a different care setting occurs. However, some professionals expressed uncertainty about role responsibilities in ACP, as the involvement of multiple disciplines occasionally created ambiguity about who should lead particular discussions: *“Of course*,* you also have the GP*,* the general practice nurse*,* and I notice that these kinds of things are discussed twice. So who is responsible? Should it be the case manager? Should it be the practice nurse? Should it be the GP? Who is going to have the conversation?”* (H6, case manager in home care).

#### Implementation leadership

Healthcare professionals noted that their readiness for engaging in ACP was supported by clear organizational leadership and reduced when such leadership was lacking within the healthcare organization. Having a designated coordinator or team can provide more structure and continuity. Currently, roles are often unclear, and dispersed responsibilities hinder ACP implementation: *“But then you also wonder*,* ‘Who will have that coordinating role? Who is going to do that?’ […] You need to have someone at the center who monitors*,* directs that process*,* collects*,* records*,* and sets out the lines of communication. And of course*,* you can make agreements with each other about this.”* (H1, nurse practitioner in nursing home). Some participants also indicated that clear policies might help: *“But if [ACP] is simply mandatory and becomes part of the care plan. And just as we now have to do these evaluations for the health insurer*,* this should actually be a mandatory element in the care plan. Yes*,* then they will do it. They’d have to*,* whereas now it still depends to some degree on the goodwill of the [healthcare professionals].”* (H11, nursing aide in home care).

#### Time to implement ACP

A lack of time to engage in ACP limited readiness, whereas having dedicated time for ACP was seen to facilitate it. ACP in dementia care was seen as time-intensive by the healthcare professionals. They emphasized the importance of dedicated time for ACP, noting that when this is built into care routines through scheduling or protected consultations, they feel more able to facilitate ACP discussions. High workloads, competing priorities, and staffing shortages were most commonly reported to lead to a lack of time to implement ACP. Time constraints were mostly reported by and related to physicians: *“The GP really has a tough time; they have the least amount of time of anyone. And yes*,* you still have to get involved*,* because ultimate responsibility lies with them. […] Well*,* I think they should be given more time. Because they are responsible*,* but they really have the least time for it*,* for themselves too.”* (H4, case manager in home care). In contrast, case managers and nurse practitioners often indicated having more flexibility to integrate ACP discussions into their care: “*However*,* I believe the case manager is perfectly capable of [taking on ACP] […] Also because we often take more time*,* of course. I mean*,* when I’m with someone*,* I often spend an hour and a half or two hours with them. Then you hopefully build up [a relationship of trust].”* (H6, case manager in home care). However, the extent to which healthcare professionals make time for ACP was also described to be influenced by the personal priority the individual healthcare professional assigns to it.

#### Procedural support

Healthcare professionals reported that procedural support, in the form of protocols, guidelines, or tools provided from organizational or national policy, would help them feel prepared to conduct ACP conversations. Lack of such support was noted to reduce healthcare professionals’ readiness. They emphasized that providing structure for the content of ACP conversations would be helpful, for example by outlining relevant ACP topics or listing possible care decisions. In addition, participants emphasized the importance of clear roles and processes, including clarity on who should be approached for ACP, which healthcare professional is responsible for initiating conversations, and how often ACP should be revisited: *“Obviously it would be helpful to have tools and to agree with each other that ‘this is the basis we are using’; and that you could use that as a tool to check off items*,* but also to indicate […] ‘that’s still open’. Then you can decide for yourself when the right time is to have the conversation.”* (H1, nurse practitioner in nursing home). Healthcare professionals further described how procedural support could guide the conduct and documentation of ACP conversations, including how to introduce ACP and where to document ACP information in the electronic health record. Especially early-career healthcare professionals were reported to benefit from clear guidance in initiating ACP: “*So that we also have a kind of manual on ‘how do you start this conversation?’ ‘Which topics?’ Especially for junior doctors or nurse practitioners*,* it’s nice to have something of a guideline.”* (H9, physician specialized in psychogeriatrics in nursing home). The lack of procedural support was reported to reduce healthcare professionals’ readiness, because they had to rely on their own judgment, which contributed to inconsistent practices, unclear roles, uncertainty about timing, and lower confidence.

## Discussion

### Main findings

This study explored factors influencing readiness for ACP in dementia from the perspectives of people with dementia, family caregivers, and healthcare professionals. Our findings demonstrate that readiness for ACP is shaped by an interplay of personal, relational, and systemic factors.

Personal factors affected readiness for ACP across all participant groups. Among people with dementia, two attitudes appeared to lower readiness: a focus on living in the present and a sense of surrendering to the uncontrollability of the future. This aligns with the socioemotional selectivity theory, suggesting that people who perceive their time as limited tend to prioritize meaningful present experiences over future planning, a perception that may be intensified following a dementia diagnosis and may affect ACP readiness [41–44]. In contrast, attitudes emphasizing autonomy and preparing for the future potentially enhance readiness. This aligns with Kukla’s concept of ‘conscientious autonomy’, where individuals seek to make value-consistent decisions and maintain a sense of control [45, 46]. In healthcare professionals, readiness was also influenced by personal priority, with those viewing ACP as a meaningful part of their day-to-day work being more intrinsically motivated to proactively initiate discussions. Readiness was further influenced by the content of ACP: people with dementia and healthcare professionals reported avoidance of sensitive or confronting or even confrontational topics, echoing prior findings that bringing up emotionally charged topics can discourage engagement [14, 20, 47–50]. However, for people with dementia, this avoidance was frequently expected to change over time, with participants anticipating a willingness to discuss certain ACP topics in the future. This suggests that people with dementia may still engage in selective future planning regarding topics they currently feel emotionally unprepared to talk about or find particularly important. More broadly, this indicates that ACP readiness should not be seen as a binary state (i.e., “ready” or “not ready”), but as a dynamic, topic-dependent concept, where an individual’s readiness may vary across ACP themes and evolve over time as circumstances and priorities change. Relational factors, reflected through social and professional support, were central to readiness among people with dementia and family caregivers specifically. Readiness was greater when the ACP process was seen as a collaborative effort, involving both the person with dementia and family caregivers and when they engaged in informal conversations. Drawing upon social support from peers to discuss personal experiences also increased readiness to engage in ACP. Readiness was further strengthened by clear and consistent information that may reduce the perceived complexity of ACP, as well as by proactive facilitation from healthcare professionals that the person with dementia and their family caregivers know or trust.

Readiness of healthcare professionals was also influenced at a system level, referring to the healthcare organization environment. Procedural support from organizational and national policy, which provides standardized guidance and clear expectations for practice, was reported to improve readiness to engage in ACP. Other system-level factors that facilitated readiness included clear leadership, dedicated time for ACP, interprofessional collaboration within and across care settings. Unclear roles and limited coordination, lack of time, a lack of interprofessional communication, and limited procedural support were reported to complicate ACP delivery, supported by previous literature [20, 21, 27, 51, 52].

### Strengths and limitations

This study has several strengths. Including people with dementia who were able to reflect on their current and future readiness for and engagement in ACP strengthened the study’s relevance by capturing their direct experiences and perspectives on readiness for ACP. In addition, family caregivers of individuals in more advanced stages of dementia contributed perspectives from later in the disease, ensuring that perspectives from across the disease trajectory were represented. The use of a qualitative design also enabled rich insights into personal experiences and perceptions, bridging theoretical concepts with practical healthcare system factors and policy implications.

A number of limitations should also be acknowledged. The participant group generally had a high educational level, no migration background, and many participating people with dementia and family caregivers had professional experience in healthcare, which may have been mixed with, or affected, their perspectives on their own readiness. The sample was probably skewed toward individuals already interested or somewhat engaged in ACP, limiting understanding of those who are more hesitant (i.e., less “ready”) or less informed.

### Implications for practice and future research

The findings have several implications for increasing readiness for ACP in practice. Presenting ACP as an ongoing, flexible process rather than a one-time decision could help reduce resistance, as it acknowledges the evolving nature of care needs and readiness and decrease pressure of having to address it all in a single conversation [53]. Incremental engagement should start with topics that individuals feel prepared to discuss, leaving room for expanding the conversation over time, adapting to changes in the individual’s circumstances, disease progression, or preferences [3, 14, 54, 55]. Consistent information provision about what ACP entails, the topics that can be addressed, and how the process may unfold over time might increase accessibility and engagement [3, 14, 27, 45]. Creating emotionally safe environments and providing multiple, low-pressure opportunities for discussing ACP topics tailored to individual coping styles and priorities is also essential.

External motivators from healthcare organizations, such as strong organizational leadership, structured procedures, and protected time for ACP discussions, may foster sustainable implementation of ACP. As structural changes within healthcare organizations can take time, parallel efforts to strengthen intrinsic motivation of individual healthcare professionals are equally important. This may include fostering a culture where ACP is openly valued as part of daily practice, encouraging healthcare professionals to share positive experiences, and reinforcing ACP as a core component of person-centered dementia care. Healthcare organizations could also encourage engagement of healthcare professionals by equipping them with tools and resources they need to facilitate ACP discussions effectively. Guidance through lists of care options, conversation prompts, and frameworks for goal-setting may help structure ACP conversations and make them more accessible for all parties involved. Moreover, enhancing interprofessional collaboration and communication within and across healthcare settings is vital to improving healthcare professionals’ readiness. Equally important is ensuring clarity about roles and responsibilities. Clearly specifying who initiates, discusses, and documents ACP may streamline communication and strengthen collaboration within and across care teams.

While this study provides important insights into factors influencing readiness for ACP in dementia, further research could examine the impact of specific interventions, such as dementia-sensitive communication training, conversation aids, or peer-support approaches, on improving readiness and confidence among people with dementia, family caregivers, and healthcare professionals.

## Conclusion

This study explored factors influencing readiness for ACP in dementia from the perspectives of people with dementia, family caregivers, and healthcare professionals. Readiness emerged as a dynamic and multifaceted concept shaped by interacting personal, relational, and organizational factors. Practical implications include creating emotionally safe and structured approaches for people with dementia and families, equipping professionals with skills and confidence to address sensitive topics, and embedding ACP within supportive organizational systems that provide leadership, time, and coordination. Addressing readiness with regard to these factors may facilitate timely and meaningful implementation of advance care planning, ensuring future care aligns with the values and preferences of people with dementia and their families.

## Supplementary Information

Appendix A. Interview guides on experiences with advance care planning, factors influencing readiness for advance care planning, and the use of conversation aids among people with dementia, family caregivers, and healthcare professionals. This appendix provides the full translated interview guides used to explore experiences with advance care planning, factors influencing readiness for advance care planning, and the use of conversation aids among people with dementia, family caregivers, and healthcare professionals.

Appendix B. Coding tree of main categories and specific sub categories describing factors influencing readiness for advance care planning among people with dementia, family caregivers, and healthcare professionals. A hierarchical coding framework showing how categories and subcategories related to readiness for advance care planning were organized during qualitative data analysis across all participant groups.

## Data Availability

The datasets used and/or analyzed during the current study are available from the corresponding author on reasonable request.

## Abbreviations

ACP: Advance care planning
GP: General practitioner
