# Supplementary material for: Factors influencing readiness for advance care planning in dementia: a qualitative interview study
Source: BMC Palliat Care. 2026 Feb 9;25:63. doi: 10.1186/s12904-026-02012-4 (PMC12983640; doi:10.1186/s12904-026-02012-4)
Supplement: Supplementary file 1 — Appendix A. Interview guides on experiences with advance care planning, factors influencing readiness for advance care planning, and the use of conversation aids among people with dementia, family caregivers, and healthcare professionals. This appendix provides the full translated interview guides used to explore experiences with advance care planning, factors influencing readiness for advance care planning, and the use of conversation aids among people with dementia, family caregivers, and healthcare professionals. [file 12904_2026_2012_MOESM1_ESM.docx]

**Appendix A.** Interview guides on experiences with advance care planning, factors influencing readiness for advance care planning, and the use of conversation aids among people with dementia, family caregivers, and healthcare professionals

**Interview guide for persons with dementia**

**Part 1: Experiences with or expectations of advance care planning**

**Introductory question:**
Since you found out that you have dementia, have you talked with doctors or nurses about the kind of care you would want if your condition were to worsen?

**If the person has experience with advance care planning:**

- Can you tell me a bit about how that conversation went?
  - Who initiated the conversation (e.g., you, or the healthcare professional)? What did you think about that?
  - Was the conversation spontaneous or planned? How did you feel about that?
- Have you recorded your preferences (for example, in an advance directive or through a notary), or do you plan to do so?
- Have there been any follow-up conversations, or are any planned?
  - If not, would you like to have one? Could you explain why or why not?

**If the participant has no experience with advance care planning:**

- Would you like to have a conversation about your wishes for future care?
  - Could you explain why or why not?
  - If yes: When would you want this to happen? Could you elaborate?
  - If yes: Would you prefer to bring it up yourself, or would you rather wait until your healthcare professional does?
- Have you talked with people close to you (e.g., family) about your preferences or about recording your wishes for future care?
- If applicable: What would you expect from such a conversation? What would you hope to achieve?

**Part 2: Factors for improving readiness for advance care planning**

**[If the participant has experience with advance care planning]**

**Factors related to healthcare professionals:**

- What did you think about the way your healthcare professional brought up the topic of your care preferences if your condition worsens? Could you explain?
  - How could they have done this better?
- Were you satisfied with how your healthcare professional conducted the conversation? Could you elaborate?
  *(e.g., clarity, empathy, openness, directness, time taken, etc.)*
  - What could have been improved?
- What did you think about the timing of the conversation? Could you elaborate?
  - Approximately how long after your diagnosis did it take place?

**Factors related to people with dementia and family caregivers:**

- What motivated you to engage in a conversation about future care?
- When did you feel ready for it?
- Did you have any concerns or doubts about discussing future care? Could you elaborate?
- If yes: Did your healthcare professional reassure you? If so, how?
- Other possible topics: feeling safe, relationship with the healthcare professional, communication style, preparation, timing, coping with diagnosis.

**[If the participant has no experience with advance care planning]**

**Factors related to healthcare professionals:**

- If you would like to have such a conversation: How could your healthcare professional best approach you about it? Could you elaborate?
- How could your healthcare professional best support you in having a conversation about your future care?
- When would you prefer to have such a conversation (e.g., soon after diagnosis or later)? Could you elaborate?
- If you do not wish to have such a conversation: Is there anything your healthcare professional could say or do to persuade you to reconsider?

**Factors related to people with dementia and family caregivers:**

- If you do not wish to have such a conversation: Could you explain why not? What holds you back?
- If you do wish to have such a conversation: What needs to happen before you would feel ready to do so?
  - What could help you feel more comfortable having a conversation about your future care needs?
- Are there things that seem difficult or that might prevent you from having such a conversation (e.g., worries or doubts)?
  - If yes: Could you explain further?
  - If yes: What could make it easier for you?
- Other possible topics: feeling safe, relationship with healthcare professional, communication style, preparation, timing, coping with diagnosis.

**Part 3: Perspectives on using conversation aids**

**Introduction to conversation aids:** [Explaining what conversation aids are and showing two examples of conversation aids; letting the participant look into them for a few minutes]

**Introductory question:**Have you ever received such a conversation aid from a healthcare professional or are you familiar with them?

- If yes: Did you use it?
- If yes: What was your experience using it?

**Main questions:**
What do you think about these kinds of tools for supporting conversations about the care wishes and needs of people with dementia and their loved ones?

- Do you think such a tool could help (or has helped) you in discussing your care preferences? Could you explain?
- Would you like to use (or use again) such a tool before your next conversation with your healthcare professional about your care preferences? Why or why not?
- If you have not yet had such a conversation: Could such a tool encourage you to have one? Why or why not?
- In what format would you prefer to receive this information?
  *(e.g., brochure, list of example questions, or more visual materials such as illustrations or images)*

**Interview guide for family caregivers**

**Part 1: Experiences with or expectations of advance care planning**

**Introductory question:**Since you found out that your loved one has dementia, have you talked with doctors or nurses about the kind of care your loved one would want if their condition were to worsen?

**If the participant has experience with advance care planning:**

- Can you tell me a bit about how that conversation went?
  - Who initiated the conversation (e.g., you, your loved one, or the healthcare professional)? What did you think about that?
  - Was the conversation spontaneous or planned? How did you feel about that?
- Has your loved one recorded their preferences (for example, in an advance directive or through a notary), or are you planning to do so?
- Have there been any follow-up conversations, or are any planned?
  - If not, would you like to have one? Could you explain why or why not?

**If the participant has no experience with advance care planning:**

- Would you like to have a conversation about your loved one’s wishes for future care?
  - Could you explain why or why not? What factors influence your opinion?
  - If yes: When would you want this to happen (e.g., now or later)? Why?
  - If yes: Would you prefer to bring it up yourself, or would you rather wait until the healthcare professional does?
- Have you talked with people close to you (e.g., family) about your loved one’s preferences or about recording their wishes for future care?
- If applicable: What would you expect from such a conversation? What would you hope to achieve?

**Part 2: Factors for improving readiness for advance care planning**

**[If the participant has experience with advance care planning]**

**Factors related to healthcare professionals:**

- What did you think about the way the healthcare professional of your loved one brought up the topic of your loved one’s care preferences if their condition worsens? Could you explain?
  - How could they have done this better?
- Were you satisfied with how the healthcare professional of your loved one conducted the conversation? Could you elaborate?
  *(e.g., clarity, empathy, openness, directness, time taken, etc.)*
  - What could have been improved?
- What did you think about the timing of the conversation? Could you elaborate?
  - Approximately how long after your loved one’s diagnosis did it take place?

**Factors related to people with dementia and family caregivers:**

- What motivated you to engage in a conversation about your loved one’s future care?
  - When did you feel ready for it?
- Did you have any concerns or doubts about discussing your loved one’s future care? Could you elaborate?
  - If yes: Did the healthcare professional of your loved one reassure you? If so, how?
- Other possible topics: feeling safe, relationship with the healthcare professional, communication style, preparation, timing, coping with diagnosis.

**[If the participant has no experience with advance care planning]**

**Factors related to healthcare professionals:**

- If you would like to have such a conversation: How could the healthcare professional of your loved one best approach you about it? Could you elaborate?
- How could the healthcare professional best support you and your loved one in having a conversation about their future care?
- When would you prefer to have such a conversation (e.g., soon after diagnosis or later)? Could you elaborate?
- If you do not wish to have such a conversation: Is there anything the healthcare professional could say or do to persuade you to reconsider?

**Factors related to people with dementia and family caregivers:**

- If you do not wish to have such a conversation: Could you explain why not? What holds you back?
- If you do wish to have such a conversation: What needs to happen before you would feel ready to do so?
  - What could help you feel more comfortable having a conversation about your loved one’s future care needs?
- Are there things that seem difficult or that might prevent you from having such a conversation (e.g., worries or doubts)?
  - If yes: Could you explain further?
  - If yes: What could make it easier for you?
- Other possible topics: feeling safe, relationship with healthcare professional, communication style, preparation, timing, coping with diagnosis.

**Part 3: Perspectives on using conversation aids**

**Introduction to conversation aids:** [Explaining what conversation aids are and showing two examples of conversation aids; letting the participant look into them for a few minutes]

**Introductory question:**Have you ever received such a conversation aid from a healthcare professional or are you familiar with them?

- If yes: Did you use it?
- If yes: What was your experience using it?

**Main questions:**
What do you think about these kinds of tools for supporting conversations about the care wishes and needs of people with dementia and their loved ones?

- Do you think such a tool could help (or has helped) you in discussing your loved one’s care preferences? Could you explain?
- Would you like to use (or use again) such a tool before your next conversation with the healthcare professional about your loved one’s care preferences? Why or why not?
- If you have not yet had such a conversation: Could such a tool encourage you to have one? Why or why not?
- In what format would you prefer to receive this information?
  *(e.g., brochure, list of example questions, or more visual materials such as illustrations or images)*

**Interview guide for healthcare professionals**

**Part 1: Experiences with or expectations of advance care planning**

**Introductory question:**Have you ever had conversations with patients with dementia and/or their family caregivers about wishes and preferences regarding future care?

**If the participant has experience with advance care planning:**

- Can you describe how you usually conduct these conversations?
- Who most often initiates these conversations or topics about care preferences and needs? (e.g., you as the healthcare professional, the patient, or the family caregiver)
- Are these conversations usually specifically planned in advance, or do they often arise spontaneously (e.g., during another discussion)?
- Do you know if discussing future care with you has led to concrete steps in recording wishes and preferences? (e.g., creating a life document, recording in the medical record, or arranging a notary appointment)
- Have there been follow-up conversations after the initial discussions, or are any planned?
  - If not, would you like to have follow-up conversations? Could you explain why or why not?
- Do you use a standard checklist or framework when discussing these topics? (e.g., based on an advance care planning guideline)
- Are there lessons you have learned over the years about conducting these conversations with patients and/or their families?

**If the participant has no experience with advance care planning:**

- Would you be willing to conduct conversations about your patients’ wishes and needs regarding future care?
  - Could you elaborate? What considerations influence your opinion?
  - What challenges or barriers exist?
- Do you discuss these topics, or conduct such conversations, if patients and/or caregivers raise them (e.g., during another discussion)?
- Could you explain how you handle such situations? What considerations guide your response?
- Does your team or supervisor discuss the implementation of advance care planning conversations regarding dementia care within your organization/department?
  - How do you view this?
  - What practical steps could be taken to integrate advance care planning into your department/organization?
- What are your expectations if you were to conduct such conversations with patients and/or caregivers? What would you want to achieve for the patient and/or their families?

**Part 2: Factors for improving readiness for advance care planning**

**[If the participant has experience with advance care planning]**

**Factors related to patients and family caregivers:**

- What contributes to patients and caregivers being ready to engage in such conversations?
- Have you ever encountered a situation where you thought a conversation about future care was needed, but the patient or caregiver did not want to discuss it?
- What, in your view, prevented them from engaging?
- How do you handle such situations? Do you wait until the patient is ready, or are there ways to encourage readiness?
- Can you provide an example where someone was initially not ready but later became ready? If so, how did this happen?
- Other possible topics: timing, safe environment, relationship with the healthcare professional, communication style, preparation, coping with diagnosis
- How do you decide the timing of such a conversation? (e.g., shortly after diagnosis, later, or variable)
- What do you need to know from the patient and/or caregiver before initiating a conversation? What should they do or say to prepare?
- In your experience, does the type of caregiver involved affect readiness (e.g., children versus partners)? Could you elaborate?

**Factors related to healthcare professionals:**

- What factors made you open to conducting these conversations?
- Have you ever felt barriers or doubts about discussing advance care planning with your patients and their families? Have you noticed this among colleagues?
  - What caused these barriers?
- What knowledge and/or skills do you need to conduct these conversations? What knowledge or skills do other healthcare professionals need, in your opinion?
- Other possible topics: time, financial resources, organizational role requirements.

**[If the participant has no experience with advance care planning]**

**Factors related to patients and family caregivers:**

- Have you observed situations where someone was very willing to talk about future care wishes?
  - Do you know what motivated them? What, in your view, contributes to their readiness?
- Have you observed situations where someone was not willing at all to discuss future care wishes?
  - What, in your view, prevented them from engaging?
- Other possible topics: safe environment, relationship with healthcare professional, communication style, preparation, timing, coping with diagnosis.

**Factors related to healthcare professionals:**

- What could make you more open to conducting these conversations? Could you explain?
- What knowledge or skills might you need to conduct advance care planning conversations effectively?
- Other possible topics: time, financial resources, organizational role requirements.

**Part 3: Perspectives on using conversation aids**

**Introduction to conversation aids:** [Explaining what conversation aids are and showing two examples of conversation aids; letting the participant look into them for a few minutes]

**Introductory question:**Are you familiar with conversation aids?

- If yes: Which ones? Do you use them in the care of your patients with dementia? Have you ever provided them to patients and/or caregivers? Do you provide them routinely? What considerations influence your use?
- How long have you been using these aids? What is your experience using them?

**Main questions:**

- What do you think about these tools for supporting conversations about the care wishes and needs of people with dementia and their caregivers?
  - What are the advantages?
  - Are there any disadvantages?
- Do you think conversation aids can encourage patients and caregivers to engage in (potentially early) conversations about care wishes and needs? Could you elaborate?
- Do you think conversation aids could encourage healthcare professionals to conduct advance care planning conversations with patients and caregivers? Could you elaborate?
- If conversation aids are not currently used: Would you consider using them in daily practice to prepare for advance care planning conversations? Why or why not?
  - If yes: What added value would they provide for you, and for patients and caregivers?
- If you have no experience with advance care planning: Could such tools motivate you to conduct conversations about advance care planning with patients and caregivers? Why or why not?
- In your view, what information is essential to include in a conversation aid to prepare patients and caregivers as effectively as possible for an advance care planning conversation?
- In what format would this information best be delivered to people with dementia and their caregivers? *(e.g., an information booklet with detailed text, or a more visual format with images or illustrations)*
