# Supplementary material for: Factors influencing readiness for advance care planning in dementia: a qualitative interview study
Source: BMC Palliat Care. 2026 Feb 9;25:63. doi: 10.1186/s12904-026-02012-4 (PMC12983640; doi:10.1186/s12904-026-02012-4)

**Appendix B.** Coding tree of main categories and specific sub categories describing factors influencing readiness for advance care planning among people with dementia, family caregivers, and healthcare professionals


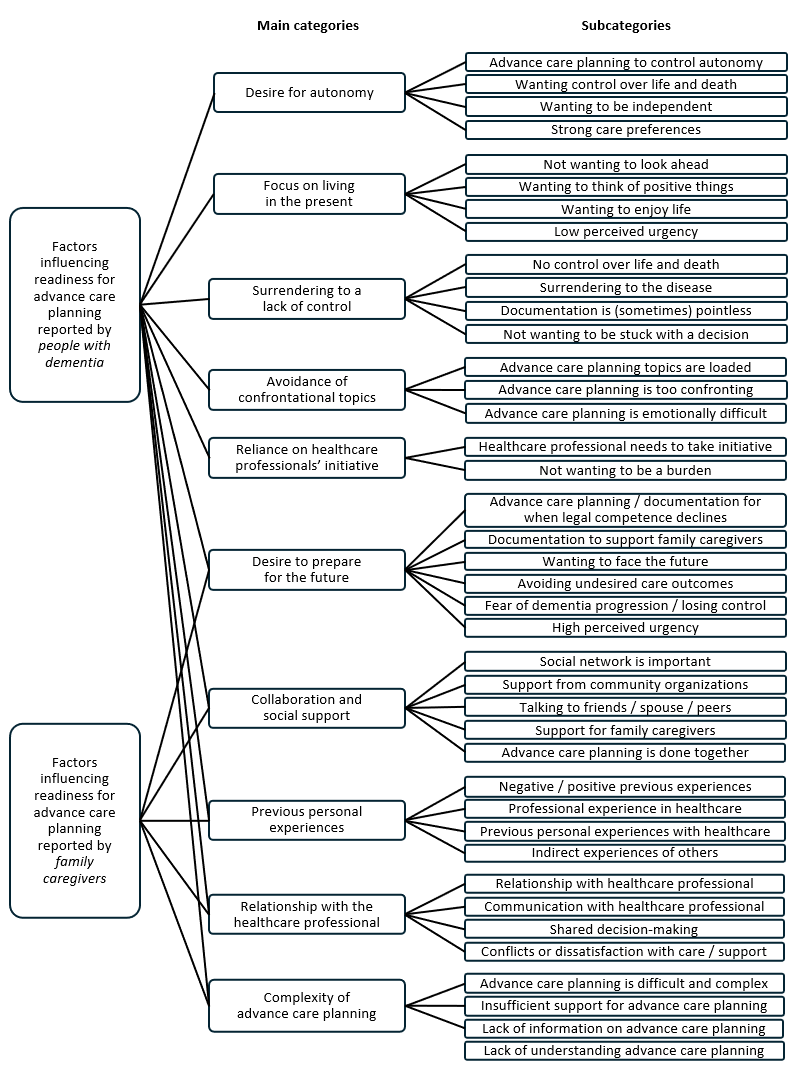


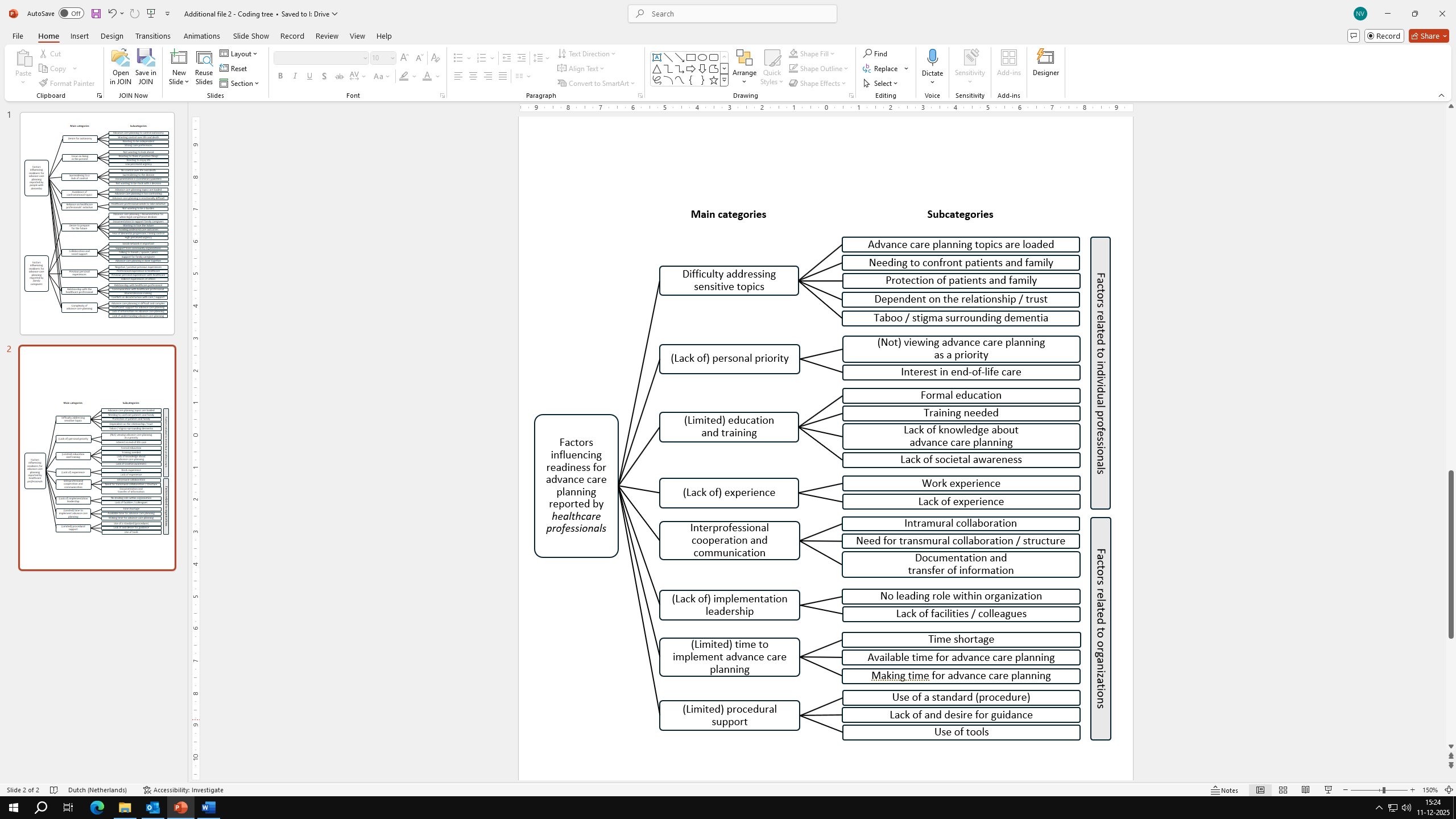

Supplement: Supplementary file 2 — Appendix B. Coding tree of main categories and specific sub categories describing factors influencing readiness for advance care planning among people with dementia, family caregivers, and healthcare professionals. A hierarchical coding framework showing how categories and subcategories related to readiness for advance care planning were organized during qualitative data analysis across all participant groups. [file 12904_2026_2012_MOESM2_ESM.docx]
